# Drug screen in patient cells suggests quinacrine to be repositioned for treatment of acute myeloid leukemia

**DOI:** 10.1038/bcj.2015.31

**Published:** 2015-04-17

**Authors:** A Eriksson, A Österroos, S Hassan, J Gullbo, L Rickardson, M Jarvius, P Nygren, M Fryknäs, M Höglund, R Larsson

**Affiliations:** 1 Department of Medical Sciences, Uppsala University, Uppsala, Sweden; 2 Department of Immunology, Genetics and Pathology, Uppsala University, Uppsala, Sweden

## Abstract

To find drugs suitable for repositioning for use against leukemia, samples from patients with chronic lymphocytic, acute myeloid and lymphocytic leukemias as well as peripheral blood mononuclear cells (PBMC) were tested in response to 1266 compounds from the LOPAC^1280^ library (Sigma). Twenty-five compounds were defined as hits with activity in all leukemia subgroups (<50% cell survival compared with control) at 10 μM drug concentration. Only one of these compounds, quinacrine, showed low activity in normal PBMCs and was therefore selected for further preclinical evaluation. Mining the NCI-60 and the NextBio databases demonstrated leukemia sensitivity and the ability of quinacrine to reverse myeloid leukemia gene expression. Mechanistic exploration was performed using the NextBio bioinformatic software using gene expression analysis of drug exposed acute myeloid leukemia cultures (HL-60) in the database. Analysis of gene enrichment and drug correlations revealed strong connections to ribosomal biogenesis nucleoli and translation initiation. The highest drug–drug correlation was to ellipticine, a known RNA polymerase I inhibitor. These results were validated by additional gene expression analysis performed in-house. Quinacrine induced early inhibition of protein synthesis supporting these predictions. The results suggest that quinacrine have repositioning potential for treatment of acute myeloid leukemia by targeting of ribosomal biogenesis.

## Introduction

During the past decades, the traditional drug discovery process has been increasingly lengthy, expensive and the rate of new drug approvals has been slow.

Thus, new strategies for drug discovery and development are needed. One such strategy is drug repositioning (or repurposing) in which a new indication for an existing drug is identified. In this approach, known on-patent, off-patent, discontinued and withdrawn drugs with unrecognized cancer activity can be rapidly advanced into clinical trials for this new indication, as much or all of the required documentation to support clinical trials can rely on previously published and readily available data.^1^

Many screening approaches for identification of new cancer drug candidates have utilized cell-free assays for detection of specific interactions with known or emerging molecular targets.^2, 3^ However, the relatively poor outcome with respect to identification of clinically novel and significantly improved cancer drugs have led to a renewed and growing interest for cancer drug screening based on compound-induced changes in cellular phenotypes.^4^ Monolayer cultures of human tumor cell lines have been the general model in these efforts. Although these are important models for prediction of mechanisms of action, they are less predictive of clinical activity.^5, 6^

Primary cultures of patient tumor cells represent an alternative tumor model system that has received very little attention in the context of cancer drug screening and development. Research efforts using primary cultures of patient tumor cell models have mainly focused on prediction of clinical activity of cancer drugs for individual patients.^7, 8^ However, non-clonogenic *in vitro* assays performed on primary cultures of patient tumor cell from different diagnoses can detect tumor-type-specific activity of standard and investigational cancer drugs^9, 10^ and has successfully been utilized in compound library screening.^11^

In the present study 12 samples of leukemia and 4 samples of normal mononuclear cells were tested in response to 1266 mechanistically annotated compounds including Food and Drug Administration-approved drugs. Quinacrine was the only compound found with activity in the three leukemia subtypes tested with concurrent low toxicity in normal mononuclear cells, and was, therefore, selected for further preclinical evaluation.

## Materials and methods

### Cell culture

#### Leukemic patient samples

Twelve samples of leukemia (four acute lymphocytic leukemia, four acute myeloid leukemia (AML), four chronic lymphocytic leukemia), as well as peripheral blood mononuclear cells (PBMC) from four healthy donors were used for the compound screen. An additional 9 AML and 10 PBMC samples were used for validation experiments. Clinical characteristics and genetic information of these nine AML patients are summarized in Table 1.

Leukemic samples were obtained by bone marrow/peripheral blood sampling. The leukemic cells and PBMCs were isolated by 1.077 g/ml Ficoll-Paque centrifugation and cryopreserved as described previously.^12^ Cell viability was determined by trypan blue exclusion test and the proportion of tumor cells in the preparation was judged by inspection of May-Grünwald-Giemsa stained cytospin slides. All samples used in this study contained >70% tumor cells. The sampling was approved by the Ethics Committee of Uppsala University (Ns 21/93 and 2007/237).

#### Cell lines

The AML cell lines HL-60 (with ability to differentiate *in vitro*),^13^ Kasumi-1 (harboring a t(8;21) mutation),^14^ KG1a (high content of immature CD34+ cells)^15^ and MV4-11 (harboring a mixed-lineage leukemia rearrangement as well as fms-like tyrosine kinase 3 internal tandem duplication)^15, 16^ were all obtained from American Type Culture Collection, ATCC, Rockville, MD, USA. The cell lines were grown in Dulbecco's Modified Eagle's Medium or Roswell Park Memorial Institute-1640 medium supplemented with 10% heat-inactivated fetal bovine serum, 2 mmol/l l-glutamine, 100 μg/ml streptomycin and 100 U/ml penicillin (obtained from Sigma-Aldrich Co, St Louis, MO, USA) and were cultured at 37 °C in humidified air containing 5% CO_2_.

### Preparation of compounds for screening

For screening, 1266 mechanistically annotated compounds, many of them previously used in the clinical setting, from the LOPAC^1280^ substance library (Sigma-Aldrich) were used. The compounds were dissolved in dimethyl sulfoxide and further diluted with phosphate-buffered saline. For further testing, quinacrine was purchased from Sigma-Aldrich. For the screening, 384-well plates were prepared with final test concentrations of 10 μM using a Biomek 2000 as described previously.^17^ For dose–response studies at seven concentrations in twofold dilutions, final test plates were prepared from source plates containing drug solutions at 10 mM using an acoustic dispensing Echo 350 instrument equipped with Labcytes Access robotics (Labcyte Inc., CA, USA).

### Measurement of drug activity

The Fluorometric Microculture Cytotoxicity Assay, described in detail previously,^18^ was used for measurement of the cytotoxic effect of library compounds. The Fluorometric Microculture Cytotoxicity Assay is based on measurement of fluorescence generated from hydrolysis of fluorescein diacetate to fluorescein by cells with intact plasma membranes. Cells were seeded in 384-well drug containing plates (screening) or blank plates (dose–response) using the pipetting robot Precision 2000 (Bio-Tek Instruments Inc., Winooski, VT, USA) and drugs were then added 12 h later using the acoustic Echo liquid dispenser.

Primary cultures of leukemic and PBMC samples were seeded at a density of 5–10 × 10^4^ cells/well. For cell lines the number of cells per well was 2500–5000, adjusted individually for each cell line. In each plate, two columns without drugs served as controls and one column with medium only served as blank. Quality criteria for a successful assay included a mean coefficient of variation of <30% in the control and a fluorescence signal in control wells of >5 × the blank. Results are expressed as Survival Index, defined as fluorescence of experimental wells in percent of unexposed control wells with blank values subtracted.

### Gene expression analysis

We followed the original protocol using HL-60 cells as described by Lamb *et al.*^19^ In brief, cells were seeded in a six-well plate at a density of 0.4 × 10^6^ cells per well. Cells were left for 24 h, followed by exposure to either quinacrine at a final concentration of 1 or 10 μM, or to vehicle control (dimethyl sulfoxide). After 6 h the cells were washed with phosphate-buffered saline and total RNA was prepared using RNeasy miniprep kit (Qiagen, Chatsworth, CA, USA). Starting from two micrograms of total RNA, gene expression analysis was performed using Genome U133 Plus 2.0 Arrays according to the GeneChip Expression Analysis Technical Manual (Rev. 5, Affymetrix Inc., Santa Clara, CA, USA).

Raw data were normalized with MAS5 (Affymetrix) and gene expression ratios for drug treated vs control cells were calculated to generate lists of regulated genes. Filter criteria were present call for all genes in the treated cell line and an expression cutoff of at least 100 arbitrary expression units. The results were uploaded to the NextBio software.

### Bioinformatic analysis using the NexBio platform

The NextBio research platform was used for bioinformatic analysis.^20^ The NextBio data mining strategy was divided into two parts. In the first part, public data were collected from diverse sources, such as NCBI GEO, Array Express, SMD, Broad Cancer Genomics, Cancer Biomedical Informatics Grid and other repositories. A data analysis step produced sets of differentially expressed gene signatures associated with each experimental or clinical comparison, such as disease versus normal. In the final step of part one, all signatures were tagged with relevant ontology terms that reflected associated tissue types, disease/phenotype, compound treatment, or genetic perturbation (for example, gene mutation, knockout, siRNA knockdown).

In the second part, rank-based enrichment statistics were applied to compute pairwise correlation scores between all signatures followed by a meta-analysis to compute individual signature-ontology concept correlation scores. After application of additional statistical criteria, such as correction for multiple hypotheses testing, the ontology concepts were ranked by statistical significance. A numerical score of 100 was assigned to the most significant result, and normalize the other results' scores to the top-ranked result, for details see ref. 20. Body Atlas is a tool that can be used to find normalized gene expression across all tissues, cell types, cell lines and stem cells in the NextBio library. A Body Atlas query for a gene expression signature or bioset will produce a list of tissues and cell types ranked by relevance. Bioset results are designated as positively or negatively correlated with a tissue or cell type. The PharmacoAtlas application was used to find compounds and treatments significantly correlated to a bioset (gene expression signature). Results are ranked in order of statistical significance. Finally, the pathway enrichment application was used to identify biogroups (gene set ontologies) for which the query signature is highly enriched.

Enrichment analysis based on gene expression results was also performed with Metadrug software (Thomson Reuters).^21^ The Metadrug database contains both public and proprietary functional ontologies that collect genes/proteins into biologically meaningful categories. In the present study the Metadrug Process ontology was used.

### Measurements of DNA and protein synthesis

Effects on DNA and protein synthesis were monitored in Cytostar-TH plates (available in the ‘*In Situ* mRNA Cytostar-TH assay' kit, Amersham International, Buckinghamshire, UK) using ^14^C-labeled thymidine and leucine. A Cytostar-TH plate has scintillants molded into the transparent polystyrene bottom. When labeled substrate is absorbed into the intracellular compartment of the cells at the bottom of the wells, the radioisotope spatially approaches the scintillant, thereby generating a detectable signal, whereas free radiolabelled substrate in the supernatant is unable to stimulate the scintillant.^22^

HL-60 cells were suspended in fresh medium containing ^14^C-thymidine (111 nCi/ml; for the DNA experiments) or ^14^C-leucine (222 nCi/ml; for the protein experiments), yielding final radioactivity in the wells of 20 and 40 nCi, respectively. Quinacrine (1–10 μM), Tween (2%) or phosphate-buffered saline was added in duplicate. Radioactivity was measured with a computer-controlled Wallac 1450 MicroBetaH trilux liquid scintillation counter (Wallac OY, Turku, Finland) immediately after addition of the cell suspension and at different time points during a 72 h cell culture period.

### Screening and dose–response data analysis and statistics

Small Laboratory Information and Management System^23^ was used for screening data management. Raw fluorescence data files were loaded into the Small Laboratory Information and Management System software, which calculates percent inhibition according to the formula: Percent inhibition=100 × (x-negative control/positive control-negative control)−1, where x denotes fluorescence from experimental wells. Small Laboratory Information and Management System also identifies and corrects systematic spatial errors. Screening data were subsequently exported to Vortex (Dotmatics Inc., Bishop's Stortford, UK) software for further analysis. More than or equal to 50% mean inhibition in the leukemic samples was set as the criterion for qualifying as hit compound.

Dose-response data were analyzed using calculated Survival Index values and the software program GraphPadPrism4 (GraphPad Software Inc., San Diego, CA, USA). Data were processed using non-linear regression to a standard sigmoidal dose–response model to obtain IC_50_-values (the concentration resulting in a Survival Index of 50%).

Analysis of data from the National Cancer Institute growth inhibitory screen in 60 cell lines (NCI-60 GI 50 data) was performed using the NCI Cellminer database (http://discover.nci.nih.gov/cellminer/).

## Results

**Figure 1:**
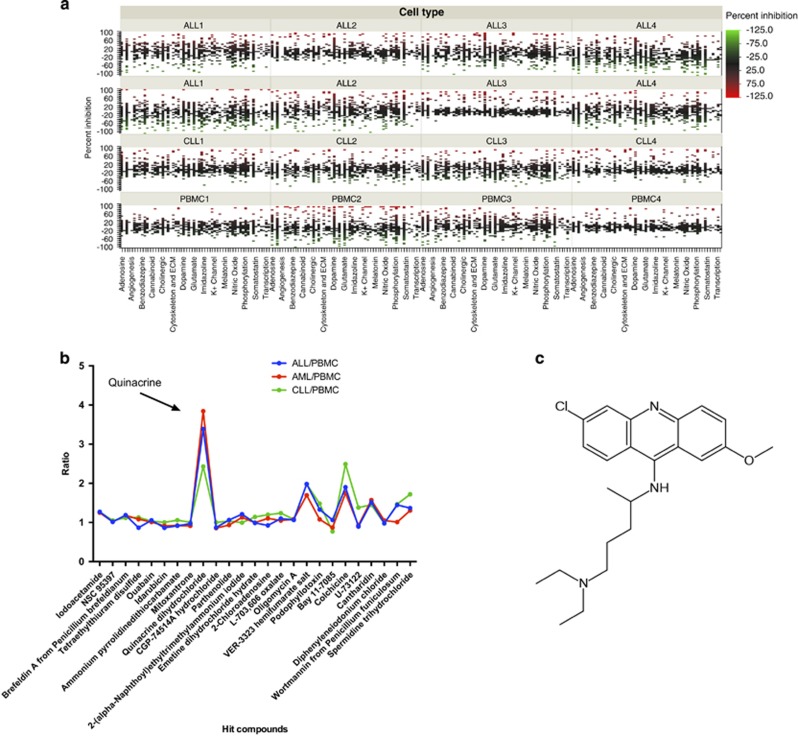
Screening the Lopac library for drug activity in a panel of leukemia (acute myeloid leukemia, acute lymphocytic leukemia, chronic lymphocytic leukemia) and PBMC cultures. The overall screening results are displayed in (**a**) and expressed as percent inhibition (PI). In (**b**) the top twenty-five hits are shown ranked as ratio of PI in PBMC/PI in the leukemic samples. The chemical structure of the top scoring hit quinacrine is shown in (**c**).

The cytotoxic/anti-proliferative activities in response to the 1266 annotated compounds from the LOPAC^1280^ library at a concentration of 10 μM in the patient leukemic cell samples and PBMCs are shown in Figure 1a. Twenty-five compounds were defined as hits, with activity in all leukemia subgroups at the screening drug concentration of 10 μM. The ratio of mean percent inhibition between the leukemia subgroups and the PBMC panel is shown in Figure 1b. Only one compound, quinacrine, showed concurrent high activity in all leukemia subgroups and low activity in normal PBMCs and was, therefore, selected for further preclinical evaluation. The chemical structure of quinacrine is depicted in Figure 1c.

**Figure 2:**
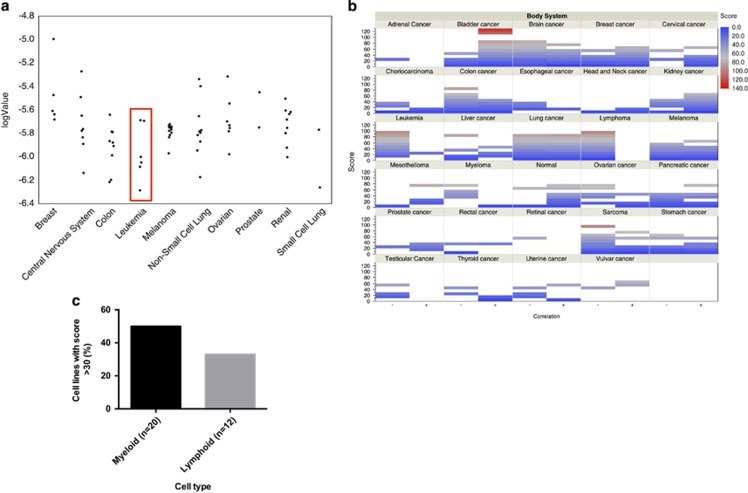
Activity of quinacrine in the NCI-60 panel expressed as log IC_50_s (**a**). In (**b**) reversibility of diagnosis-specific gene expression ranked by reversibility score using the body atlas app in NextBio is shown. Positive correlations are indicated as + and negative correlations as − for 567 cell lines grouped into different tumor types as indicated. In (**c**) the fraction of myeloid and lymphoid cell lines with negative reversibility scores exceeding 30 is shown.

Mining the NCI-60 database demonstrated pronounced anti-leukemia activity for quinacrine (Figure 2a). Querying the NextBio cell line panel database for the ability of quinacrine to reverse diagnosis-specific gene expression revealed leukemias to have the highest reversibility score (Figure 2b). Separating the leukemia cell lines into those of myeloid and lymphocytic origins, the former showed a higher frequency with reversibility scores above 30 in the NextBio body atlas application (Figure 2c).

**Figure 3:**
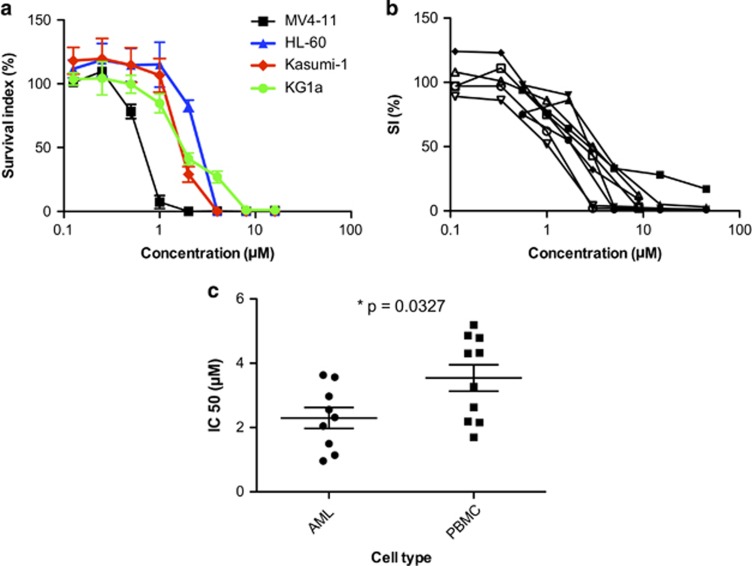
Dose-dependent effects of quinacrine on cell survival in the four AML cell lines indicated (**a**). Survival was determined over 72 h using the FMCA assay. The results are expressed as percentage of the untreated control and presented as mean values±s.e.m. from three independent experiments. In (**b**) dose-dependent effects of quinacrine on different primary AML cultures from patients (*n*=9) are shown. The results are expressed as survival index (%) determined by the FMCA. In (**c**) IC_50_ is compared for AML (*n*=9) and PBMC (*n*=10) using Student's *t*-test (*P*<0.05, two-tailed test).

Subsequently, we validated the screening results by dose–response testing in four AML cell line models as well as in nine additional patient samples of acute myeloid leukemia (Table 1). In the cell line panel, the fms-like tyrosine kinase 3-mutated and mixed-lineage leukemia-rearranged MV4-11 cells were the most sensitive with an IC_50_ of 0.63 μM followed by Kasumi-1, KG1a and HL-60 with IC_50_ values of 1.93, 1.97 and 2.24 μM, respectively (Figure 3a). In the patient AML samples, quinacrine showed activity in the low μM range with a mean IC_50_ of 2.30 μM (Figure 3b), statistically significantly lower than that of normal PBMCs; 3.54 μM (Figure 3c; *P*=0.0327; Student's *t*-test).

**Figure 4:**
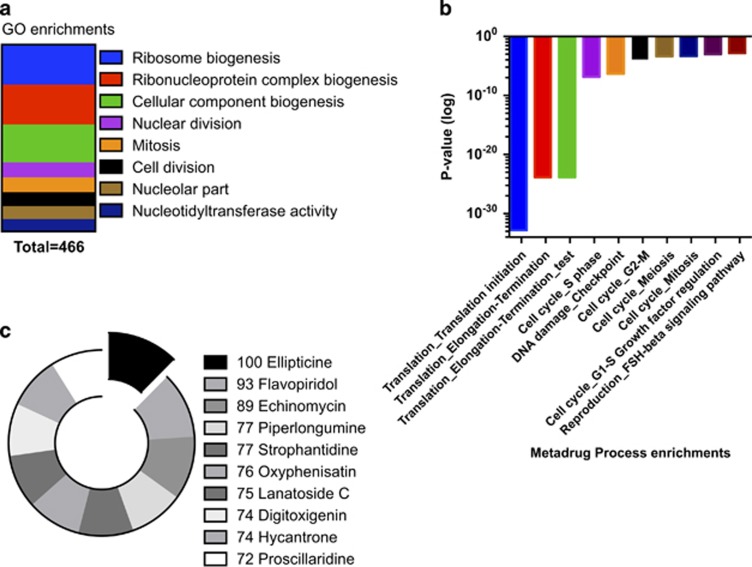
Gene set enrichment analysis using NextBio GO ontology scores (**a**) and Metacore Process ontology (**b**). In (**c**) the Pharmaco-atlas of NextBio to rank the drugs inducing the most similar gene expression signatures as quinacrine (**c**).

**Figure 5:**
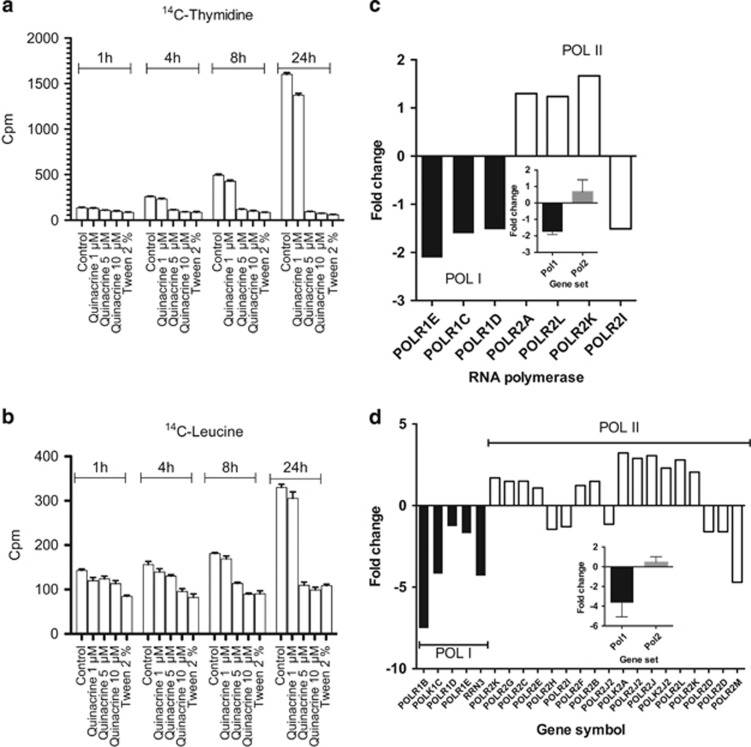
Effect of quinacrine on DNA (**a**) and protein synthesis (**b**). In (**c**) NextBio was used to retrieve genes associated with RNA polymerase I and II (pol-I and pol-II) based on quinacrine induced gene expression results in HL-60 present in the database. The results are expressed as fold change from vehicle treated controls. In (**d**) an in-house gene expression analysis of HL-60 cells exposed to quinacrine was performed and imported into NextBio and analyzed as in (**c**).

Next, mechanistic exploration was performed in the NextBio bioinformatic software using gene expression analysis of drug-exposed myeloid leukemia cultures (HL-60) present in the database. Gene enrichment analysis revealed strong connections to ribosomal biogenesis nucleoli and translation initiation (Figure 4a). This was supported by a parallel gene set enrichment analysis performed using the Metadrug pathway analysis software (Figure 4b). The highest drug–drug correlation retrieved from the NextBio pharmaco-atlas application was to ellipticine (Figure 4c). Interestingly, ellipticine has indeed been found to be a selective RNA polymerase I (pol-I) inhibitor.^24^ Quinacrine also induced early inhibition of both DNA and protein synthesis (Figures 5a and b) supporting these predictions. To further investigate this hypothesis, we used the NextBio to retrieve genes associated with pol-I and RNA polymerase II (pol-II) based on quinacrine-induced gene expression results in HL-60 present in the database. An apparent decrease in the expression of several pol-I-associated genes was observed while three out of four of the retrieved pol-II genes were upregulated (Figure 5c). To substantiate these observations we performed an in-house gene expression analysis of HL-60 cells exposed to quinacrine and imported the results into NextBio. A very similar pattern was observed with downregulation of most retrieved pol-I associated genes, whereas pol-II genes were mostly upregulated (Figure 5d).

## Discussion

In the present study, unbiased screening in primary cultures of human leukemias and PBMCs in response to a mechanistically annotated compound library identified quinacrine as an active agent. The use of primary cultures of human tumor cells from patients rather than commonly used cell lines may have distinct advantages by mimicking the clinical situation more closely.^7, 8^ Indeed good correlations with clinical outcome have been observed using these model systems.^7, 25^ Quinacrine appeared most active in AML, which consequently emerge as a candidate diagnosis for quinacrine treatment.

Quinacrine is an acridine derivative, which was developed in the early 1920s and used extensively as an antimalarial during the Second World War. It was used by over three million soldiers for up to 4 years in a controlled setting, making it one of the most extensively studied drugs ever. Quinacrine has also been used for the treatment of giardiasis and shows therapeutic activity against several autoimmune disorders such as systemic lupus erythematosis and rheumatoid arthritis.^26^ Quinacrine is no longer commercially available in the United States or European Union but is still marketed in parts of Asia.

Although quinacrine has been shown to exert antitumor activity in several cell line models of solid tumors, this is the first paper to show significant activity also in leukemia. This was not only demonstrated in AML cell lines but primarily in more clinically relevant cell cultures obtained directly from patients. Quinacrine stands out as an attractive candidate for repositioning as an anti-leukemic agent because of the wealth of existing data about its safety, tolerance and pharmacokinetics available.^26, 27, 28^

Quinacrine intercalate into double-stranded DNA, which is considered to be an important primary target for the observed antitumor activity.^28^ However, in contrast to many other intercalating drugs, quinacrine does not induce DNA damage and is not considered to be mutagenic in human cells.^28^ Several other mechanisms of action potentially related to the interaction with DNA have been postulated including inhibition of topoisomerase II^29^ and NF-kappaB^30^ as well as activation of the p53 signal pathway.^31, 32^ Moreover, quinacrine have a profound effect on the cellular transcription machinery, particularly targeting cell stress-related transcription. Thus, quinacrine inhibits heat shock factor-1,^33^ a major transcriptional regulator of the unfolded protein response and hypoxia inducible factor 1-α, a transcription regulator that promotes tumor cell survival under the conditions of limited oxygen supply.^28^ Furthermore, quinacrine has been shown to interact with phospholipid bilayers leading to inhibition of phospholipase A2 and C, which in turn may affect many membrane-spanning channels and transporters that require the presence and correct architecture of phospholipids.^28^ One example is the blockage of P-glycoprotein.^34^

In the present paper, the bioinformatic analysis pointed toward pol-I as a potential molecular target mediating quinacrine activity. This was further strengthened by measurements of gene expression changes induced after quinacrine treatment. Pol-I genes were downregulated, whereas pol-II genes were considerably less affected. Indeed, inhibition of pol-I could possibly explain and contribute to some previously described actions of quinacrine such as topoisomerase II inhibition and p53 activation. Blockers of topoisomerase I and II activity have been reported to disrupt pol-I transcription.^35^ In particular, the ellipticine drug family, which has demonstrated antitumor activity in clinical trials, was previously proposed to be the result of DNA breakage following the formation of an ellipticine–topoisomerase II–DNA complex.^36^ However, recently ellipticine was shown to specifically inhibit pol-I transcription.^24^ In this context, it is notable that the present data show that quinacrine induces gene expression with highest correlations to that of ellipticine. Also, following inhibition of ribosome biogenesis, several ribosomal proteins are released from the nucleolus, which bind MDM2 and inhibit its ubiquitin ligase activity toward p53, resulting in p53 accumulation.^35^

Interestingly, quinacrine was also found to downregulate expression of the Myc oncogene (sevenfold, not shown), which is a known activator of pol-I transcription.^35^ Therefore, quinacrine can potentially target ribosome biogenesis through both direct inhibition of ribosomal DNA transcription by DNA intercalation and downregulation of expression of transcriptional activation of Myc, resulting in enhanced antiproliferative activity especially in malignant cells expressing a high level of the Myc protein. Indeed, overexpression of Myc has been reported to be associated with adverse clinical outcome and drug resistance in AML.^37, 38^ However, definitive conclusions about quinacrine being a selective pol-I inhibitor will require further more detailed molecular studies.

Although the wide use of quinacrine demonstrates that it is a generally safe compound, some side effects have been identified. The most common adverse effects associated with quinacrine are dizziness, headache and gastrointestinal disturbances such as nausea and vomiting^26^ (http://www.micromedexsolutions.com). Reversible yellow discoloration of the skin, conjunctiva and urine may occur during long-term use or after large doses. High doses used in the treatment of giardiasis may occasionally cause transient acute toxic psychosis reversible upon cessation of quinacrine^26^ (http://www.micromedexsolutions.com). Other rare but serious side effects of the compound are different forms of dermatitis and aplastic anemia. These side effects develop gradually, and are reversible if quinacrine is discontinued. Hepatitis and hepatic necrosis occur rarely^26^ (http://www.micromedexsolutions.com).

The typical route of quinacrine administration is oral but it can also be administrated by other routes. It is rapidly absorbed from the gastrointestinal tract with plasma levels increasing 2–4 h after administration and reaching a peak in 8–12 h. Plasma concentration increases during the first week and reaches steady-state by the fourth week. The plasma levels of quinacrine are low in comparison with tissue concentrations. Peak plasma concentrations of up to 0.32 μM for quinacrine have been documented on a standard malaria regimen.^26^ The highest concentrations are found in the liver, spleen, lungs and adrenal glands. Interestingly from a repositioning perspective, quinacrine also accumulates in white blood cells reaching intracellular concentrations exceeding 23 μM.^27^ The major route of quinacrine elimination is via the renal system.^26^

In conclusion, the overall results indicate that quinacrine, being a multi-targeted agent with a favorable safety profile have repositioning potential for treatment of AML and should be further evaluated in a clinical trial context in this disease.

## Figures and Tables

**Table 1:** Acute myeloid leukemia patients (*n*=9) in the dose–response experiments (clinical characteristics, molecular genetics status and karyotype)

*Age (years)*	*Sex*	*Status*	*Subclass (FAB)*	*Karyotype*	*Molecular genetics FLT3-ITD/FLT3-TKD/NPM1/CEBPA/wt*
21	F	*De novo*	M2	46 XX, del (16)(q22q22)	wt
70	M	Relapse	M0	47XY, +13(13)/46 XY(10)	FLT3-ITD mutation
26	F	*De novo*	M0	46 XX	wt
54	F	Relapse	M2	46 XX	NPM1 mutation
68	F	*De novo*	M1	Complex incl del 5q & del 17p	wt
78	M	*De novo*	M1	46 XY	wt
42	M	*De novo*	M0	Complex incl del 5q & del 17p	wt
62	M	*De novo*	M0/M1	46 XY	wt
22	M	*De novo*	M4	46 XY	CEBPA biallelic mutation

Abbreviations: CEBPA, CCAAT/enhancer-binding protein alpha; F, female; FAB, French-American-British; M, male. FAB indicates French-American-British classification; complex karyotype indicates karyotype with three or more aberrations. The molecular characterization include analysis for mutations in fms-like tyrosine kinase 3 (FLT3; internal tandem duplication mutations (FLT3-ITD) and point mutations in the tyrosine kinase domain (FLT3-TKD), nucleophosmin 1(NPM1) and CCAAT/enhancer-binding protein alpha (CEBPA; biallelic or monoallelic mutation). Wild type (wt) indicates the absence of mutations in FLT3, NPM1 and CEBPA.

## Notes

RL, PN and MF are co-founders and shareholders of Repos Pharma AB, a small Swedish research and development company dedicated to investigations of drug repositioning in the cancer area. The remaining authors declare no conflict of interest.
